# A full-spectrum aqueous extract of black cardamom (*Amomum subulatum*) improves focus/alertness and executive function: a randomized, double-blinded, placebo- and active-controlled, comparative study in healthy working-class participants

**DOI:** 10.3389/fnins.2026.1786880

**Published:** 2026-04-08

**Authors:** Jestin V. Thomas, M. E. Mohan, Syam S. Das, Nithyanandam Allimuthu, Sheena Devasia, P. A. Aneesa, Krishnakumar Illathu Madhavamenon, Baby Chakrapani Pulikkaparambil Sasidharan

**Affiliations:** 1 Leads Clinical Research and Bio Services Private Limited, Bangalore, India; 2 BGS Global Institute of Medical Sciences, Bengaluru, India; 3 R&D centre, Akay Natural Ingredients Private Limited, Kochi, India; 4 Tamil Nadu Government Multi Super Specialty Hospital, Chennai, India; 5 Centre of Neuroscience, Department of Biotechnology, Cochin University of Science and Technology, Kochi, India; 6 Department of Biochemistry, Sree Sankara College, Kochi, India; 7 Centre of Excellence in Neurogeneration and Brain Health, Kochi, India

**Keywords:** *Amomum subulatum*, black cardamom, caffeine, CNS vital signs, cognition, large cardamom, nootropics

## Abstract

**Background:**

Interest is increasing in natural, plant-based, food-grade nootropics that deliver sustained cognitive benefits without adverse effects. Caffeine remains the most widely consumed psychoactive substance, owing to its rapid, short-term enhancement of wakefulness, alertness, and overall performance. The present study investigated the nootropic effect of a full-spectrum aqueous extract of black cardamom fruits and its influence when combined with caffeine.

**Methods:**

A randomized, double-blind, placebo- and active-controlled four-arm study assessed the acute cognitive effects of full-spectrum black cardamom extract (MA2-24) in comparison with caffeine. Ninety-six healthy adults (35–65 years) were randomized (*n =* 24/group) to receive placebo, MA2-24 (250 mg), caffeine (200 mg), or their combination (MA2-24 + caffeine). Cognitive performance was assessed at baseline and at 1-, 3-, 5-, and 8-h post-dose using CNS vital signs assessments.

**Results:**

MA2-24 significantly improved reaction time, accuracy, and error rate in the Shifting Attention Test and increased correct responses while reducing errors in the Symbol Digit Coding Test. The Stroop Test also showed significant improvement in reaction time and a reduction in the number of errors. Effects were significant versus baseline and placebo, mainly through 3-h post-dose, and were comparable to caffeine. Co-supplementation of MA2-24 with caffeine produced greater and more sustained benefits, especially during the later phase (5–8-h post-dose).

**Conclusion:**

The results indicate that black cardamom fruit extract MA2-24 exhibits nootropic properties across multiple domains of attention, working memory, and processing speed to support cognitive flexibility and executive function. When combined with caffeine, it showed the potential to augment and prolong caffeine’s functional effects on mental performance and fatigue management.

**Clinical trial registration:**

https://ctri.nic.in/Clinicaltrials/pmaindet2.php?EncHid=MTE0NzIx&Enc=&userName=, identifier CTRI/2024/09/074472

## 1 Introduction

Memory, thinking, attention, learning, and problem-solving represent the fundamental cognitive domains that support information processing, behavioral adaptation to dynamic environments, executive functions, and decision-making. Brain fog, or mental fatigue, is a condition that can impair these key cognitive domains, ultimately affecting an individual’s ability to sustain concentration, process information, and productivity (Kunasegaran et al., 2023; Bufano et al., 2024). In today’s technology-driven world, the pursuit of enhanced productivity has increased the demand for effective strategies to mitigate cognitive dysfunction and support optimal mental performance (Suliman et al., 2016). In this context, commonly recommended strategies including nutritious food, yoga, meditation, cognitive training, and dietary supplements/nutraceuticals, especially to manage sleep, stress, digestion, hydration, etc., and have received great attention globally (Livingston et al., 2020).

Nootropics are a class of compounds, commonly derived from foods and botanical sources such as plant extracts and phytonutrients, that may enhance cognitive function. These compounds may support alertness, attention, learning, and memory, while generally lacking the significant adverse effects associated with traditional stimulants (Malík and Tlustoš, 2022). Nootropics may act generally by (1) modulating cerebral energy metabolism, or (2) enhancing cholinergic neurotransmission, or (3) regulating excitatory amino acid receptor activity, and/or (4) modulating steroid hormone responsiveness (Mondadori, 1994). Commonly consumed nootropics include caffeine, L-theanine, *Bacopa monnieri*, *Ginkgo biloba*, ashwagandha, and omega-3 fatty acids, among many others.

Caffeine, a methylxanthine derivative widely distributed in tea and coffee, is the most widely studied food-grade nootropic agent, famous for its rapid and short-term cognitive benefits, particularly its effects on wakefulness and alertness, leading to heightened vigilance, improved encoding speed, and faster performance in semantic memory tasks (Hewlett and Smith, 2006). However, it can cause addiction, jitters, energy crashes, or anxiety, particularly at higher doses. Cessation in habitable consumers may experience a “caffeine crash,” or “caffeine withdrawal,” which is characterized by symptoms such as headaches, excessive tiredness, irritability, and hence the inability to concentrate/focus (Temple et al., 2017). Excessive caffeine intake, typically beyond 400 mg per day, has also been associated with a range of adverse effects, including disturbances in cardiovascular function, calcium homeostasis, and bone metabolism (Silva-Cavalcante et al., 2013; Temple et al., 2017; Adıgüzel and Köroğlu, 2022). Other commonly reported side effects include nervousness, irritability, headache, palpitations, sleep disturbances, nausea, dizziness, and tremors (Temple et al., 2017). Consequently, there is a growing interest in natural, caffeine-free, plant-based alternatives that support cognitive performance without undesirable effects.

Cardamom, both black cardamom/large cardamom (*Amomum subulatum* Roxb.) and green cardamom (*Elettaria cardamomum*), are widely used culinary spices and have gained attention in this context. Traditionally, cardamom was used in the Indian and Chinese systems of medicine for its nootropic and therapeutic properties (Kumar Singhal et al., 2022). Recent research has also demonstrated the beneficial pharmacological effects of these cardamom species and their phytocompounds and essential oils, especially the antioxidant, anti-inflammatory, and neuroprotective effects (Juergens, 2014; Kandikattu et al., 2017; Auti and Kulkarni, 2019; Saini et al., 2021; Abdel-Rasoul et al., 2023; Alruhaili et al., 2023; Ballester et al., 2023; Gazmer et al., 2023; Hoch et al., 2023; Bala et al., 2024). Given the importance of the emerging evidence supporting the effects of black cardamom and its bioactive constituents on brain functions, the present study aimed to evaluate the cognitive benefits, particularly mental energy, attention, focus, and processing speed, of a standardized aqueous extract of black cardamom (commonly known as Indian cardamom), hereafter referred to as MA2-24, in healthy human volunteers for the first time. The study was conducted in comparison with caffeine following a randomized, double-blind, placebo- and active-controlled, comparative design.

## 2 Materials and methods

### 2.1 Study material

Dried fruits of black cardamom (*A. subulatum* Roxb.) were received from selected farms in Northeast India and were identified by an authenticated botanist following macroscopy, microscopy, DNA analysis, and high-performance thin-layer chromatography (HPTLC, CAMAG, Muttenz, Switzerland). A voucher specimen (AKB-AS-03/24) was deposited at the Herbarium at Akay Bioactives Innovation Centre, Cochin, India. The test substances, the standard aqueous extract of MA2-24 (Batch No: ASEU-010-SC-01/24, Date: 08/09/24) and the natural caffeine from *Coffee arabica* beans (Batch No: CCAE-950-HC-02/24, Date: 18/04/24), were produced in the good manufacturing plant (GMP) facility of Akay Natural Ingredients, Cochin, India, following their proprietary production protocols. The extracts were accompanied by a certificate of analysis and a food safety data sheet indicating suitability for human intervention.

The test substances were prepared as visually identical 3 g sachets using predefined formulations. Briefly, the placebo consisted of 500 mg of sucralose and 2.5 g of tapioca maltodextrin. The caffeine sachet contained 200 mg of caffeine, 500 mg of sucralose, and 2.3 g of tapioca maltodextrin. The MA2-24 sachet consisted of 250 mg of black cardamom fruit extract powder, 500 mg of sucralose, and 2.25 g of tapioca maltodextrin. The co-supplementation sachet contained 200 mg of caffeine, 250 mg of black cardamom fruit extract powder, 500 mg of sucralose, and 2.05 g of tapioca maltodextrin. All components were homogeneously blended before being packed into sachets.

### 2.2 Ethical considerations

Good Clinical Practice (GCP) standards and the ethical principles outlined in the Declaration of Helsinki and the clinical research guidelines of the Government of India were followed to conduct the study. The protocol received approval from a registered Institutional Ethics Committee and was subsequently registered with the Clinical Trial Registry of India (CTRI/2024/09/074472, dated 27/09/2024).

### 2.3 Sample size calculation

*A priori* power analysis was conducted using G* Power for a repeated-measures ANOVA (within–between interaction). Assuming a medium effect size (Cohen’s d *f* = 0.25), alpha = 0.05, power = 80%, four treatment groups, and five time points, a minimum total sample size of 84 participants was required. Allowing for approximately 10–15% attrition, 96 participants (24 per group) were enrolled to ensure adequate statistical power to detect clinically meaningful differences in cognitive performance.

### 2.4 Study design and protocol

**Figure 1:**
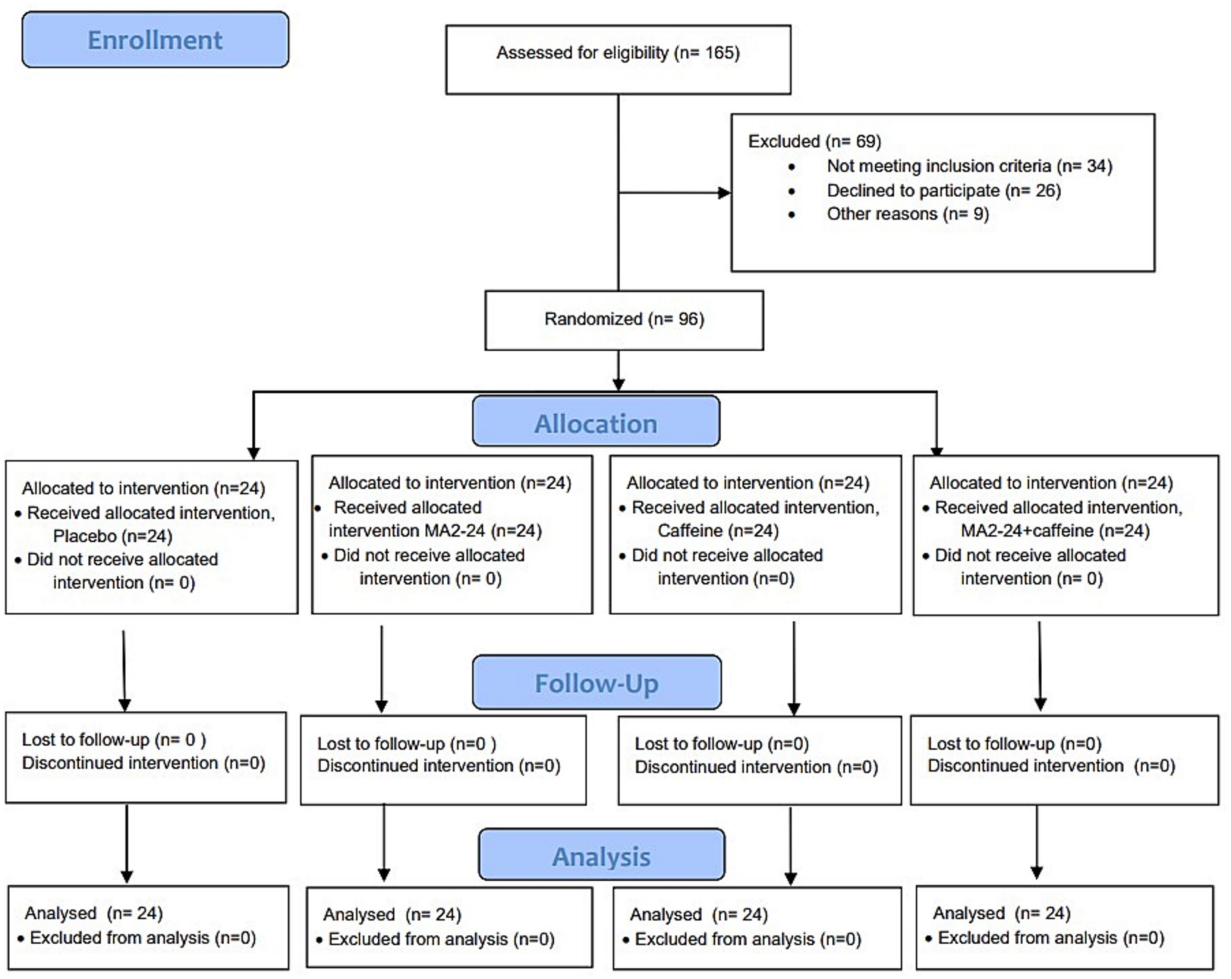
CONSORT flow diagram shows participant enrollment, allocation, follow-up, and analysis, together with the schematic representation of the study’s cohort design.

A randomized, double-blind, placebo- and active-controlled, four-arm, parallel-group comparative design was used for the study, which consisted of placebo, MA2-24, caffeine, and their combination, and assessed the cognitive performance using the central nervous system vital signs (CNSVS) tests, designed to evaluate the cognitive performance and physiological parameters in working professionals. A total of 96 participants (59 males and 37 females) who met the inclusion and exclusion criteria (Supplementary Table S1) and were aged between 35 and 65 years were enrolled after obtaining written informed consent. On the screening day (visit 1), all demographic details, physical examination, anthropometric measurements, medical history, and vital signs, including systolic and diastolic blood pressure, heart rate, and body temperature, were recorded (Supplementary Tables S2, S3). Participants reported a habitual caffeine intake of approximately two servings of coffee/tea per day. However, they were instructed to refrain from caffeine or stimulant-containing products for at least 48 h before the intervention. Volunteers were randomly assigned to one of the four intervention groups (*n =* 24 per group) using a permuted-block randomization scheme. Participants received identical sachets containing either a placebo (250 mg; Group I), MA2-24 (250 mg; Group II), caffeine (200 mg; Group III), or a combination of MA2-24 (250 mg) and caffeine (200 mg; Group IV). All participants underwent baseline assessments under fasting conditions. A standardized meal plan, including breakfast, lunch, evening snack, and water *ad libitum*, was followed. The investigational products (IPs) were administered 30–45 min post-breakfast as identical sachets dissolved in 200 ± 20 mL of water. Vital signs and cognitive performance using the CNSVS test battery were assessed at baseline and at 1-, 3-, 5-, and 8-h post-IP administration. Adverse events (AEs) were recorded throughout the study. The participant flow and cohort design of the trial are illustrated in Figure 1.

#### 2.4.1 CNS vital signs (CNSVS)

CNSVS is a validated, computerized neurocognitive assessment platform designed to evaluate multiple domains of brain function with high sensitivity and reliability. It is widely used to detect subtle changes in neurocognitive performance over time and is therefore a valuable tool for evaluating therapeutic interventions (Gualtieri and Johnson, 2008). In the present study, the Shifting Attention Test (SAT), the Symbol Digit Coding (SDC) Test, and the Stroop Test (ST) were used for primary evaluation.

SAT was used to assess executive function, cognitive flexibility, and response inhibition. It measured participants’ ability to shift between cognitive sets, apply rule-based categorization, and make rapid decisions. The test provided outcomes including the number of correct responses, errors, and reaction times, which collectively reflected cognitive agility and decision-making efficiency (Hany et al., 2025). The SDC test evaluated complex attention, visual-perceptual processing speed, and information processing accuracy. Participants matched symbols to digits using a reference key, and outcomes included the number of correct responses and errors. This test captured both the speed and precision with which participants processed and responded to visual stimuli (Huppert et al., 2017). ST was used to assess cognitive inhibition, disinhibition, and executive control. It generated outcome measures such as simple and complex reaction times (correct), Stroop-specific reaction times (correct), and commission errors. This test was sensitive to frontal lobe dysfunction and served as a robust indicator of inhibitory control and higher-order executive processing (Scarpina and Tagini, 2017).

### 2.5 Safety and tolerance

Safety and tolerability were evaluated through continuous monitoring of AEs, physical examinations, and serial measurements of vital signs. Baseline assessments were conducted in the fasting state prior to breakfast, and safety parameters were monitored throughout to identify any acute physiological changes or adverse reactions to the IPs.

### 2.6 Statistical analysis

All statistical evaluations were performed using IBM SPSS Statistics for Windows, Version 28.0 (IBM Corp., Armonk, NY, USA). Data were expressed as delta mean ± standard deviation (SD). Normality of the distribution was assessed using the Shapiro–Wilk test. Within-group comparisons at different time points were analyzed using Student’s paired *t*-test. To evaluate the treatment effects across groups and time, a repeated-measure multivariate analysis of variance (MANOVA) was performed using a 4 × 4 factorial design, with the Bonferroni correction applied for multiple comparisons. A *p*-value of < 0.05 was considered statistically significant at a 95% confidence interval. Effect sizes were calculated to interpret the clinical relevance of statistically significant findings. Demographic variables, including age, height, weight, and BMI, were analyzed using one-way ANOVA to confirm the absence of significant baseline differences among the four groups.

## 3 Results

### 3.1 Materials

**Figure 2:**
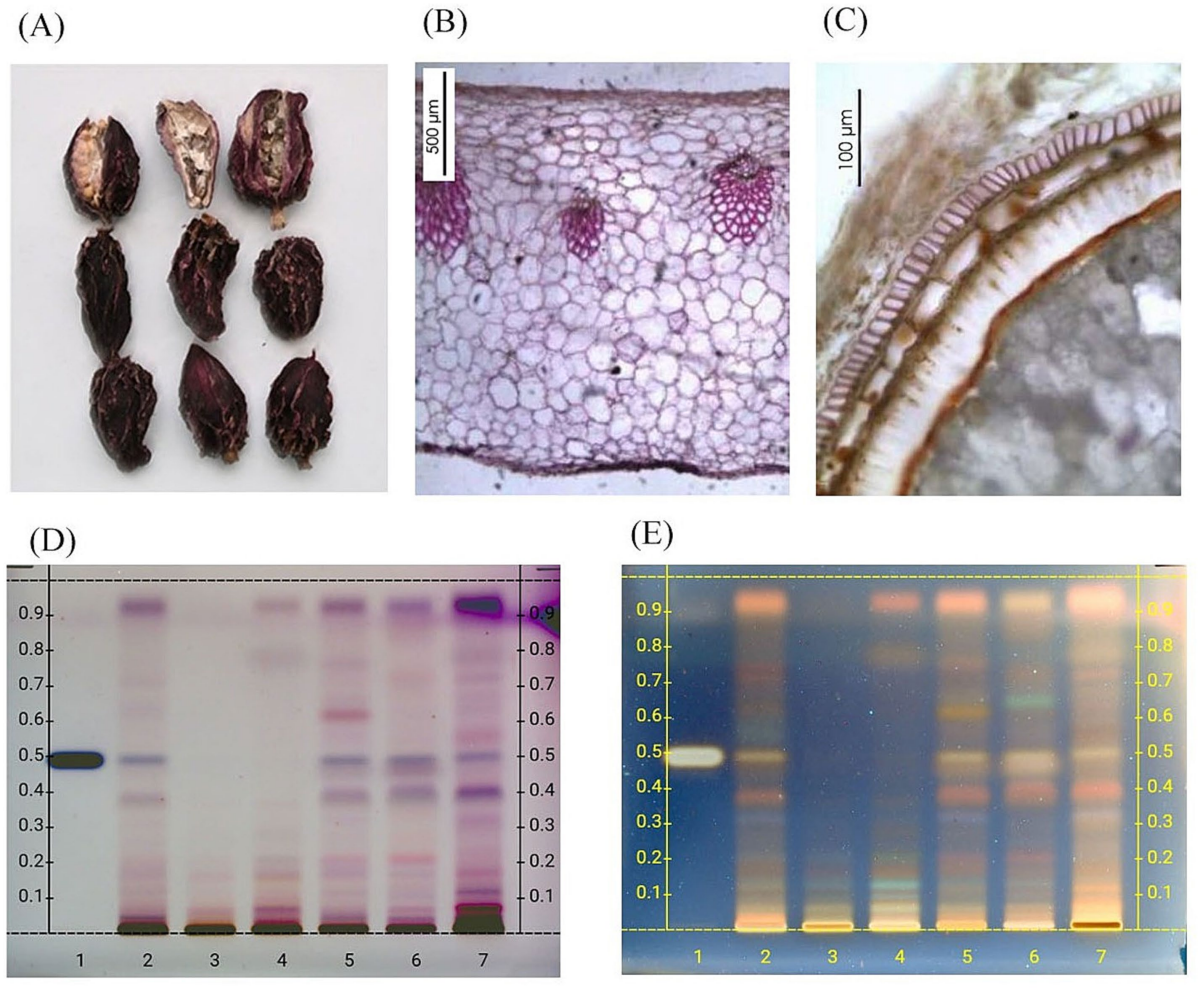
Characterization of dried *A. subulatum* fruits used for the preparation of MA2-24 extract used in the present study. **(A)** Macroscopic evaluation/digital photograph, **(B,C)** microscopic evaluation, **(D,E)** HPTLC analysis with photo-documentation under UV light at 254 nm and under fluorescence at 366 nm, respectively. Lane 1 contained *β*-sitosterol in methanol; lanes 2 and 3 contained different reference samples of *A. subulatum* fruit. Lane 5 corresponded to the test sample (*A. subulatum* raw material). Lane 6 is the reference standard of *Amomum kravanh* fruit, and lane 7 represents the reference sample of *Elettaria cardamomum* (fruit).

Macroscopy, microscopy, DNA, and HPTLC analyses of the raw material used for the preparation of the extract MA2-24 confirmed it as dried fruits of *A. subulatum* Roxb. (Figure 2). Digital photographs revealed that the seeds were mostly irregularly ovoid, with three flattened faces, externally covered by a colorless membranous aril. The seeds appeared pinkish-brown to dark brown in color and exhibited a characteristic aromatic odor with a spicy, pungent taste. Microscopic analysis revealed a very thin membranous aril composed of several layers of collapsed cells containing oil globules and prismatic oxalate crystals. The testa consisted of a single-layered epidermis of rectangular cells, followed by one to two layers of collapsed, thin-walled parenchymatous cells. The perisperm was composed of polygonal, thin-walled parenchymatous cells containing round to oval starch grains. Further DNA analysis showed 100% homology with reference sequences available in the National Centre for Biotechnology Information (NCBI)^1^ database. HPTLC analysis, compared with a standard reference sample, also confirmed the identity of the sample as *A. subulatum.*

### 3.2 Demographic and clinical characteristics

A total of 96 participants were enrolled in the study following the screening procedures, with a mean (± SD) age of 43.94 ± 6.4 years. The cohort consisted of 61% male and 39% female working professionals, providing gender representation. The anthropometric characteristics of the participants revealed a mean (± SD) body weight of 68.40 ± 9.87 kg, a mean height of 166.22 ± 8.06 cm, and a mean BMI of 24.70 ± 2.64 kg/m^2^. These parameters were statistically analyzed to ensure homogeneity across the four study arms, with no significant differences observed, confirming appropriate randomization. Vital signs, including systolic blood pressure (SBP), diastolic blood pressure (DBP), heart rate (HR), and body temperature, were closely monitored at baseline and at multiple time points.

### 3.3 CNSVS outcomes

#### 3.3.1 SAT

**Figure 3:**
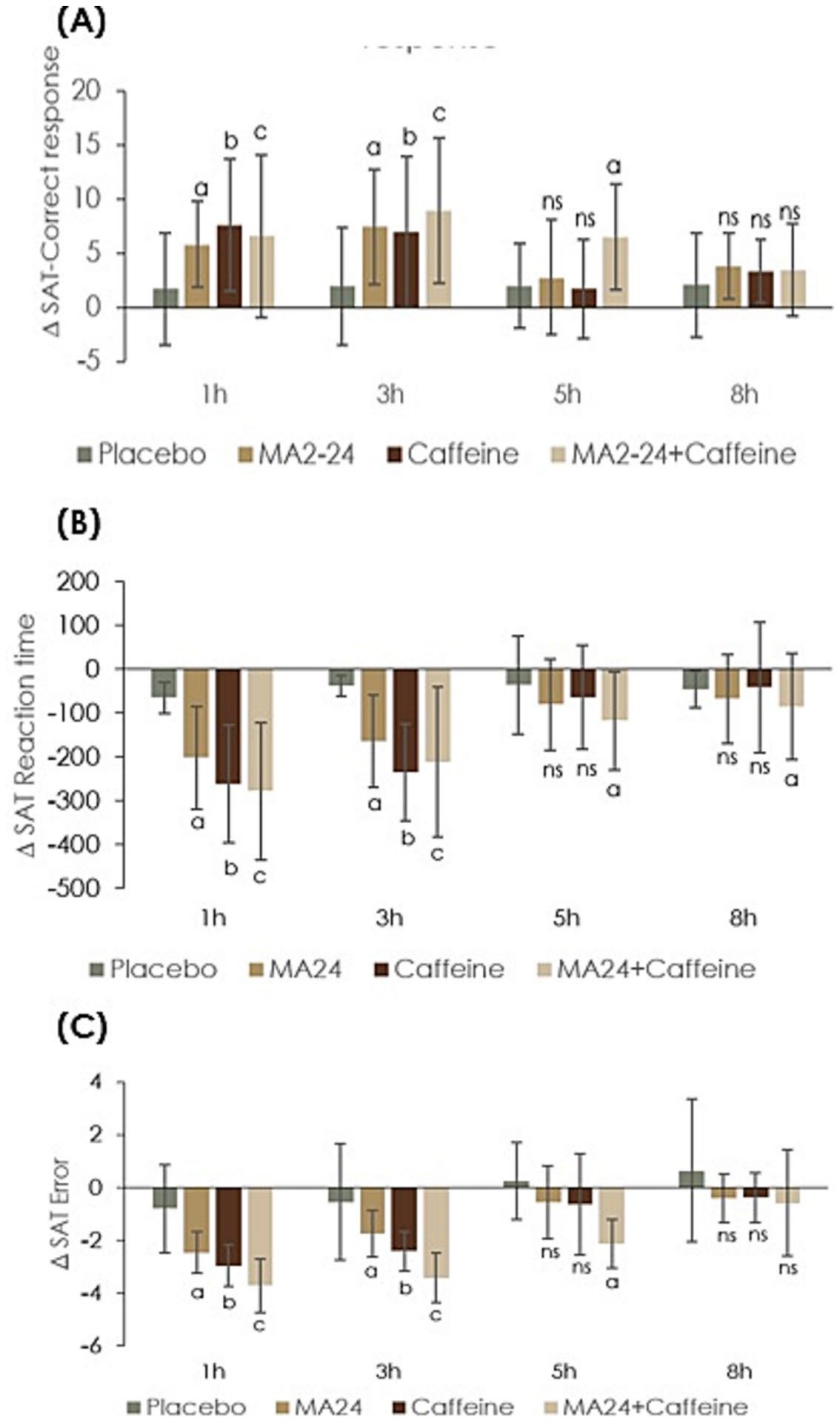
CNS vital signs–shifting attention test (CNSVS-SAT) outcomes showing changes from baseline. **(A)** Number of correct responses, **(B)** reaction time, and **(C)** number of errors across treatment groups and assessment time points. Data were expressed as *Δ* (delta) mean ± standard deviation (SD). Within-group comparisons at different time points were analyzed using Student’s paired *t*-test. Treatment effects across groups and time were evaluated by repeated-measure multivariate analysis of variance (MANOVA) using a 4 × 4 factorial design, with Bonferroni correction applied for multiple comparisons. A *p* < 0.05 was considered statistically significant at 95% confidence interval. The letters a, b, and c indicate statistically significant differences compared with placebo, while “ns” denotes non-significant results.

Repeated measures MANOVA of the difference in the number of correct responses (*Δ*cr) on the SAT revealed a statistically significant treatment effect, with an effect size of 0.50 (*F* = 5.85, *p* = 0.002). The time effect size was 0.60 (*F* = 17.3, *p* < 0.001) and the treatment × time interaction effect size was 0.76 (*F* = 2.85, *p* = 0.003), indicating a positive effect of treatment with MA2-24 (Table 1). The “Δ” increased significantly in MA2-24, caffeine, and in the co-supplemented group at 1- and 3-h post-administration time points, compared to placebo. The observed “Δ” was non-significant in the 5- and 8-h post-administration points for both MA2-24 and caffeine (*p* > 0.05). Between-group analysis of Δ correct responses revealed no significant differences among the three test substances at the 1- and 3-h time points; however, the co-supplementation group demonstrated a significant effect at 5 h when compared to placebo (Figure 3A).

**Table 1:** CNS vital data points.

Parameters	Subdomains	Groups	Δ 1 h	Δ 3 h	Δ 5 h	Δ 8 h	*p*-value
Shifting Attention Test	Number of correct responses	Placebo	1.7 ± 5.17	1.95 ± 5.4	2.04 ± 3.92	2.08 ± 4.79	T = 0.002 t = <0.001 T^x^t = 0.003
		MA2-24	5.83 ± 3.97	7.45 ± 5.31	2.79 ± 5.33	3.83 ± 3.08	
		Caffeine	7.62 ± 6.07	7 ± 7	1.7 ± 4.6	3.33 ± 2.92	
		MA2-24 + Caffeine	6.58 ± 7.53	9 ± 6.73	6.54 ± 4.86	3.45 ± 4.3	
	Reaction Time	Placebo	−64.7 ± 36.6	−38.04 ± 23.36	−35.45 ± 111.36	−45.33 ± 42.88	T = <0.001 t = <0.001 T^x^t = <0.001
		MA2-24	−201.16 ± 116.82	−163.54 ± 104.11	−80.37 ± 103.76	−67.2 ± 101.46	
		Caffeine	−261.25 ± 134.55	−234.5 ± 110.96	−63.91 ± 117.66	−40.7 ± 149.11	
		MA2-24 + Caffeine	−277.58 ± 155.59	−210.2 ± 170.54	−117.08 ± 112.08	−85.87 ± 120.99	
	Errors	Placebo	−0.79 ± 1.66	−0.54 ± 2.2	0.25 ± 1.45	0.66 ± 2.71	T = <0.001 t = <0.001 T^x^t = 0.024
		MA2-24	−2.45 ± 0.77	−1.75 ± 0.89	−0.54 ± 1.38	−0.41 ± 0.92	
		Caffeine	−2.95 ± 0.78	−2.41 ± 0.75	−0.62 ± 1.91	−0.37 ± 0.94	
		MA2-24 + Caffeine	−3.71 ± 1.02	−3.41 ± 0.93	−2.14 ± 0.92	−0.57 ± 2.01	
Symbol Digit Coding	Number of correct responses	Placebo	2.58 ± 6.47	−1.29 ± 7.86	−2.25 ± 6.26	−1.12 ± 7.79	T = <0.001 t = <0.001 T^x^t = <0.001
		MA2-24	8.29 ± 9.68	6.5 ± 7.13	2 ± 9.69	0.45 ± 8.09	
		Caffeine	10.12 ± 4.75	9.25 ± 4.6	1.12 ± 5.73	−1.79 ± 3.9	
		MA2-24 + Caffeine	14.04 ± 4.37	11.79 ± 3.94	12 ± 4.19	1.33 ± 4.55	
	Errors	Placebo	−0.12 ± 1.15	0.33 ± 0.96	0.08 ± 0.92	0.29 ± 1.04	T = <0.001 t = <0.001 T^x^t = <0.001
		MA2-24	−0.91 ± 0.92	−1.12 ± 0.79	−0.29 ± 0.75	−0.2 ± 0.77	
		Caffeine	−1.5 ± 0.72	−1.66 ± 1.09	−0.33 ± 0.76	−0.12 ± 0.94	
		MA2-24 + Caffeine	−1.54 ± 0.83	−1.45 ± 0.93	−0.75 ± 0.98	0.12 ± 0.94	
Stroop Test	Stroop test	Placebo	34.83 ± 87.67	24.37 ± 105.07	−8.41 ± 94.46	−15.87 ± 112.1	T = 0.034 t = <0.001 T^x^t = <0.001
		MA2-24	−40.87 ± 20.15	−34.54 ± 28.03	−21.91 ± 20.29	−9.04 ± 33.83	
		Caffeine	−74.5 ± 61.89	−62.58 ± 23.3	−15.87 ± 34.97	−16.58 ± 29.38	
		MA2-24 + Caffeine	−86.75 ± 82.84	−67.66 ± 82.87	−73.7 ± 107.25	−4.2 ± 37.75	
	Simple test	Placebo	−30 ± 83.39	−25 ± 72.15	−40.54 ± 73.52	27.62 ± 56.55	T = <0.001 t = <0.001 T^x^t = <0.001
		MA2-24	−83.54 ± 33.55	−66.33 ± 23.5	−48.16 ± 68.23	23 ± 51.84	
		Caffeine	−92 ± 33.56	−79.79 ± 41.37	−25.62 ± 56.08	30.25 ± 33.18	
		MA2-24 + Caffeine	−142.29 ± 27.99	−127.54 ± 30.32	−107.75 ± 33.8	24.66 ± 96.31	

The table presents Δ (delta) values for CNS vital signs outcomes. Δ represents the mean change from baseline to the specified time point. Abbreviations: T, treatment; t, time; T^×^t, treatment-by-time interaction.

Regarding SAT reaction time (RT), a significant reduction from baseline was observed in all treated groups compared with placebo. Mixed ANOVA analysis of the mean change in RT from the baseline (Δrt) showed significance with a treatment effect size of 0.82 (*F* = 15.21, *p* < 0.001), time effect size 0.88 (*F* = 58.25, *p* < 0.001), and treatment × time effect, 0.93 (*F* = 12.66, *p* < 0.001) (Table 1). Though MA2-24 and caffeine significantly reduced the RT compared to placebo (*p* < 0.05), caffeine showed better reduction (Δrt) than MA2-24 at 1- and 3-h post-ingestion time points (*p* < 0.001). The co-supplemented group, on the other hand, showed statistically significant Δrt at 5- and 8-h post-administration time points (*p* < 0.05), indicating a sustained effect (Figure 3B).

Mixed ANOVA analysis of the difference in the mean number of errors (Δerr) showed significant treatment (effect size: 0.75, *F* = 41.65, *p* < 0.001), time (effect size 0.87, *F* = 41.31, *p* < 0.001), and treatment × time effect (effect size: 0.66, *F* = 2.97, *p* = 0.024) (Table 1). Both the MA2-24 and caffeine groups significantly reduced the “number of errors” compared to placebo (*p* < 0.05) at the 1- and 3-h time points. However, there were no significant differences (*p* > 0.05) between these groups at the 3-h post-ingestion time point. Further, only the co-supplemented group showed a significant effect at the 5-h post-administration time point (Figure 3C).

#### 3.3.2 SDC

**Figure 4:**
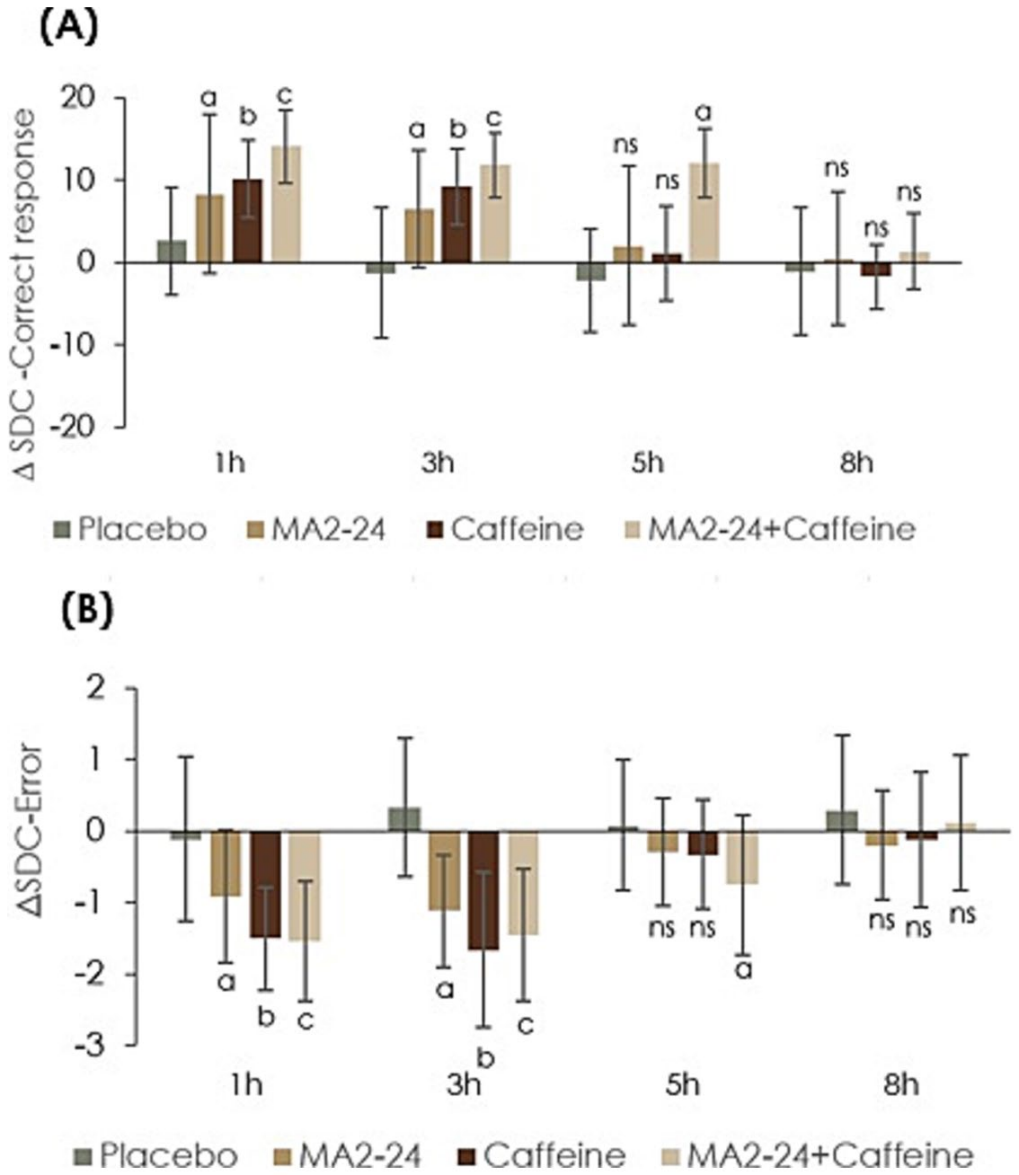
CNS vital signs–symbol digit coding (CNSVS-SDC) test outcomes showing changes from baseline. **(A)** Number of correct responses and **(B)** number of errors across assessment time points. Data were expressed as Δ (delta) mean ± standard deviation (SD). Within-group comparisons at different time points were analyzed using Student’s paired *t-t*est. Treatment effects across groups and time were evaluated by repeated-measure multivariate analysis of variance (MANOVA) using a 4 × 4 factorial design, with Bonferroni correction applied for multiple comparisons. A *p* < 0.05 was considered statistically significant at a 95% confidence interval. The letters a, b, and c indicate statistically significant differences compared with placebo, while “ns” denotes non-significant results.

The results of the SDC Test showed a significant treatment effect (effect size 0.73, *F* = 17.29, *p* < 0.001), time effect (effect size 0.91 F= 45.12, *p* < 0.001) and treatment × time effect (effect size 0.84 F= 8.40, *p* < 0.001) when the change in the number of correct responses from the baseline (Δcr) was compared with the placebo (Table 1). Both MA2-24 and caffeine significantly increased Δcr compared to placebo (*p* < 0.05), at the 1- and 3-h time points, with a slightly better effect for the caffeine group than for MA2-24; however, they were not significant at the 5- and 8-h time points. Co-supplementation usage showed a sustained and significant effect up to 5-h post-administration (Figure 4A).

Similarly, a significant reduction in the difference in the number of errors from baseline (Δerr) was observed in all three test groups, compared to placebo. The treatment effect size was 0.60 (*F* = 13.21, *p* < 0.001), the time effect size was 0.91 (*F* = 57.47, *p* < 0.001), and the treatment × time interaction effect size was 0.90 (*F* = 7.72, *p* < 0.001) (Table 1). MA2-24 and caffeine significantly reduced the Δerr compared to placebo (*p* < 0.05) at the 1- and 3-h time points, with no significant differences between the groups (*p* > 0.05). Co-supplementation showed significantly better results, especially at the 5-h post-time point, indicating a longer effect (Figure 4B).

#### 3.3.3 ST

**Figure 5:**
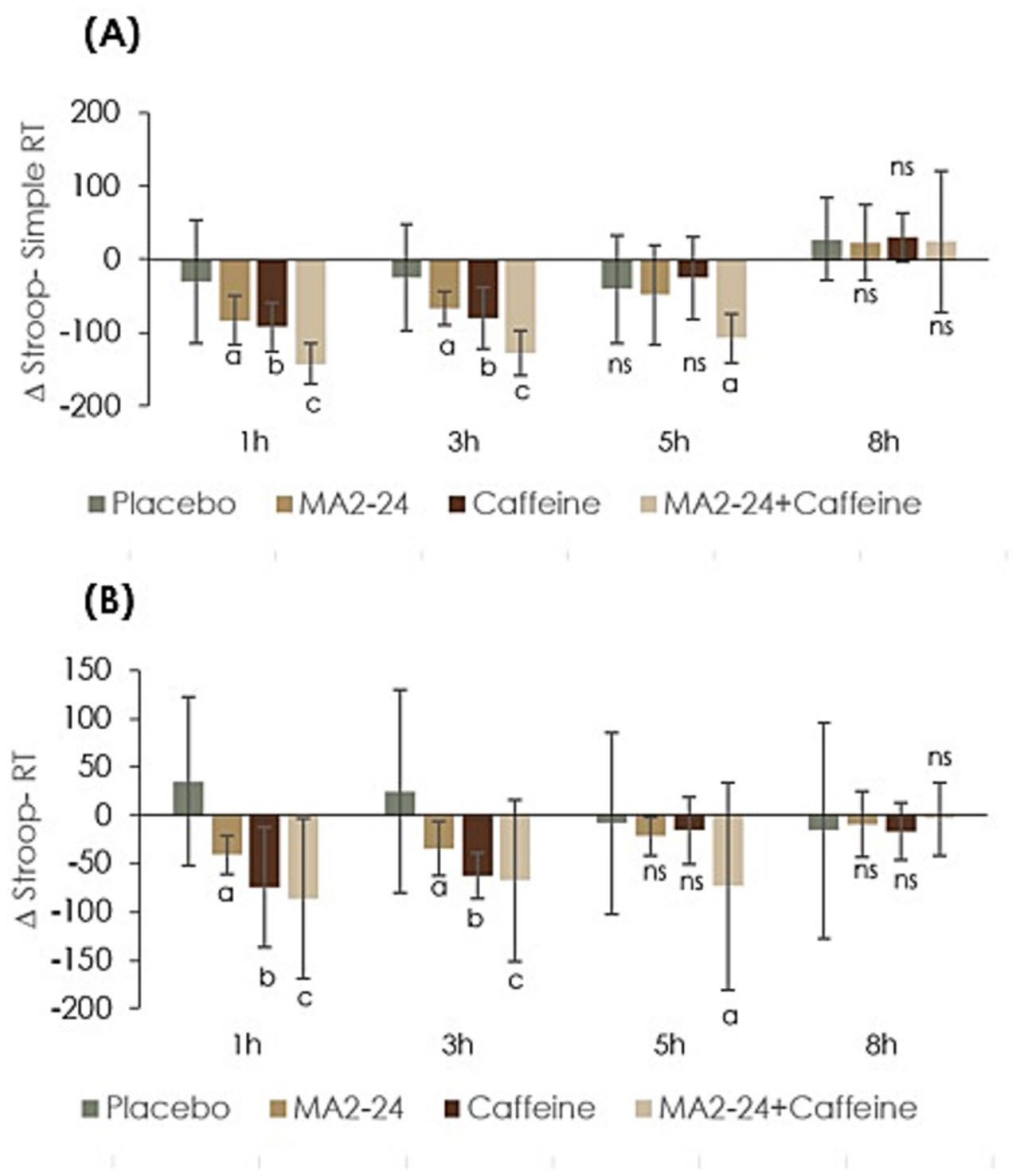
CNS vital signs–Stroop test outcomes show changes from baseline. **(A)** Simple reaction time and **(B)** Stroop reaction time points. Data were expressed as Δ (delta) mean ± standard deviation (SD). Within-group comparisons at different time points were analyzed using Student’s paired *t-t*est. Treatment effects across groups and time were evaluated by repeated-measure multivariate analysis of variance (MANOVA) using a 4 × 4 factorial design, with Bonferroni correction applied for multiple comparisons. A *p-*value of < 0.05 was considered statistically significant at a 95% confidence interval. The letters a, b, and c indicate statistically significant differences compared with placebo, while “ns” denotes non-significant results.

A statistically significant reduction in Stroop simple RT was observed in all the treatment groups, compared to both their respective baseline and the placebo. The observed treatment effect size, when the difference in RT from the baseline (Δrt) across all the treatment groups was analyzed, was 0.58 (*F* = 10.55, *p* < 0.001), the time effect size was 0.96, *F* = 191.08, *p* < 0.001), and the treatment × time interaction effect size was 0.81 (*F* = 7.07, *p* < 0.001) (Table 1). MA2-24 significantly reduced simple RT compared to baseline and placebo (*p* < 0.05) at the 1- and 3-h time points, and the observed effect was similar to caffeine, though it exhibited a better effect. Co-supplementation showed better reduction in RT, and the effect was significant up to the 5-h time point (*p* < 0.05) (Figure 5A).

A significant reduction was also observed for Stroop-Stroop RT across the treatment groups. The observed treatment, time, and treatment × time effect sizes were, respectively, 0.33 (*F* = 5.41, *p* = 0.034), 0.70 (*F* = 10.36, *p* < 0.001), and 0.93 (*F* = 14.08, *p* < 0.001) (Table 1). MA2-24 and caffeine significantly reduced Stroop-Stroop RT at the 1- and 3-h time points (*p* < 0.05). The co-supplementation group, on the other hand, showed better reduction than the caffeine group at the 1-, 3-, and 5-h time points, indicating a synergetic and sustained effect (Figure 5B).

### 3.4 Safety and tolerance

Safety and tolerability were systematically assessed *via* physical examinations and participant-reported outcomes. The investigational product MA2-24 was well tolerated, with no clinically significant abnormalities observed during post-intervention assessments. However, 72% of participants in the caffeine group reported discomfort, including increased heart rate (*n =* 6), gastrointestinal disturbance (*n =* 4), sleep disturbance (*n =* 4), and increased diuresis (*n =* 2), which were not seen in the MA2-24 group. There was a significant reduction in the number of participants (27%) and in the severity of discomfort in the co-supplemented group. The main issues observed were increased heart rate (*n =* 2), increased urination (*n =* 2), and gastrointestinal disturbance (*n =* 2). The outcomes of all AEs were noted.

## 4 Discussion

Improving and/or maintaining executive functions relies heavily on core skills such as cognitive flexibility, attentional control, and processing speed to adapt to changing situations. Caffeine, the most popular psychoactive nootropic substance and a food component, is consumed worldwide with a record of approximately 120,000 metric tons per year (Weinberg and Bealer, 2001; Laurent et al., 2014). At a dose of 200 mg, caffeine can act as a potent central nervous system stimulant primarily by crossing the blood–brain barrier and antagonizing all four adenosine receptor subtypes (A1, A2a, A2b, and A3) (Evans et al., 2024). Antagonization of receptors A1 and A2A results in increased neurotransmitters such as dopamine, glutamate, acetylcholine, norepinephrine, and serotonin, leading to heightened alertness, focus, and improved memory/performance (Ribeiro and Sebastiao, 2010; Rodak et al., 2021). However, its effects peak within an hour and decline sharply (Srivastava et al., 2017). Moreover, high doses can induce caffeine crash, leading to significant fatigue, anxiety/ nervousness, headache, and mood disturbances, limiting its sustained use, especially for sensitive individuals (Rodak et al., 2021). This highlights the need for alternatives or strategies, particularly natural nootropics that can provide sustained cognitive benefits without the negative consequences.

In this context, natural nootropic alternatives to conventional stimulants such as caffeine are gaining increasing attention, especially the simple aqueous extracts of food components with favorable organoleptics and safety for their potential to support mental energy and performance. Though many herbal extracts have shown some effects in certain domains of brain health functions, none of them have exhibited a true caffeine-like effect (Malík and Tlustoš, 2022). Recently, *Alpinia officinarum* has been reported to improve mental energy parameters, including alertness, reaction time, and focus (Eraiah et al., 2023).

The present study evaluated the cognitive potential of MA2-24, a proprietary aqueous extract of black cardamom fruit, and its combination with caffeine using validated CNSVS tests. Prior studies have shown the effectiveness of CNSVS tests in comprehensively assessing neuropsychological performance in chronic clinical conditions and in evaluating the impact of therapeutic or nutritional interventions (Campman et al., 2017; Lanou et al., 2023). MA2-24 (250 mg) alone showed significant improvements on SAT, SDC, and Stroop tests, comparable to 200 mg caffeine, though to a lesser extent.

Comparison of the mean change in SAT reaction time from baseline with that of placebo at 1- and 3-hour post-administration time points was significant (*p <* 0.05), but was not significant at 5- and 8-hour time point (*p* = 0.331) for MA2-24, indicating the absence of a longer duration effect. A similar observation was also made in the caffeine group. However, co-supplementation was also significant at both 5- and 8-h time point (*p* < 0.05). In the case of SAT errors, the change in errors from the baseline for both MA2-24 and caffeine groups was significant (*p* < 0.05) at 1- and 3-h but was not significant at 5- and 8-h time point (*p* = 0.07) compared to placebo. Co-supplementation, on the other hand, showed significance up to 5-h post-ingestion time period (*p* < 0.05). A similar comparison of the change in number of correct responses from baseline was significant for all the three groups, MA2-24, caffeine and co-supplementation groups, at 1 and 3 h (*p* < 0.05), but was not significant at 5- and 8-h time points (*p >* 0.05), except for the co-supplemented group which showed significance at 5-h time point (*p* < 0.05). The above results indicate a probable synergistic action of MA2-24 with caffeine, extending its effect for a longer duration.

Comparison of the mean change in SDC correct response from baseline with that of placebo at 1- and 3-h post-administration time points was significant (*p* < 0.05), but was not significant at 5- and 8-h time points (*p >* 0.5) for MA2-24 and caffeine groups. Co-supplementation showed significance at 1-, 3-, and 5-h time points (*p* < 0.05), but was not significant at 8 h. In the case of SDC errors, the change in errors from the baseline for all three groups also followed the same trend as the SDC correct response. Co-supplementation, on the other hand, showed significance up to an 5-h post-ingestion time period (*p* < 0.05). These results indicate improved SDC precision and accuracy by both MA2-24 and caffeine for the initial 1- to 3-h, though caffeine had a superior effect compared to MA2-24. The combination of MA2-24 with caffeine, on the other hand, produced the largest gains, with significant sustained effects particularly in the later phase (5-h post-dose).

A similar trend was observed in Stroop test parameters, simple reaction time, and Stroop reaction time, where MA2-24 and caffeine significantly reduced these parameters compared to placebo at 1 and 3 h, while caffeine produced stronger effects initially. However, caffeine had no effect at 5- and 8-h post-ingestion. Co-supplementation resulted in greater reductions in both reaction times (*p* < 0.05), which sustained through 5 h. Thus, the improvements in reaction times reflected gains in processing speed, selective attention, and executive functioning (Scarpina and Tagini, 2017). Collectively, the results of SAT, SDC, and ST imply the improvement in mental energy with MA2-24, and its possible synergetic action with caffeine to extend the effect of caffeine for a longer duration.

The sustained window of action observed with MA2-24 is consistent with its documented antioxidant and anti-inflammatory effects, which may reduce central fatigue, stabilize neuronal firing, and prevent performance deterioration over time. The cognitive and alertness-enhancing contribution of caffeine when combined with MA2-24 can be explained by its rapid neurochemical actions and its ability to modulate the activity of co-administered phytochemicals (Kennedy and Wightman, 2022). Caffeine quickly increases neuronal excitability through adenosine-receptor antagonism and elevates key neurotransmitters (dopamine, norepinephrine, serotonin, and acetylcholine) via inhibition of monoamine oxidase and acetylcholinesterase (Kennedy and Wightman, 2022). These mechanisms underpin improvements in attention, vigilance, and working memory. Additionally, MA2-24 prolongs the effects of caffeine, probably by the interaction of phytochemicals in black cardamom that downregulate CYP1A2, thereby slowing caffeine metabolism (Belayneh and Molla, 2020).

The cognitive benefits observed with MA2-24, particularly in executive functioning, may be attributed to its rich full-spectrum phytochemical composition. The extract mainly contains saponins (>25% w/w) along with polyphenols and flavonoids (~2%), including gallic acid, catechins, quercetin, and luteolin, alkaloids (1800 mg/kg), and terpenoids; mainly 1,8-cineole (~ 2.5%). These bioactive constituents are already recognized for their antioxidant, anti-inflammatory, and neuroprotective properties (Kabir et al., 2023; Dinata et al., 2024). Polyphenols and terpenoids from *A. subulatum* have been shown to enhance synaptic plasticity via upregulation of brain-derived neurotrophic factor (BDNF) signaling, improve cerebral blood flow, and modulate dopaminergic and cholinergic neurotransmission—mechanisms central to attention, working memory, and processing speed, which help in executive function (Auti and Kulkarni, 2019; Colombage et al., 2024). Such effects were also clear from the study of a methanolic extract of *A. subulatum,* which promoted dopaminergic neuron regeneration and restored dopamine levels (Gazmer et al., 2023). Additionally, 1,8-cineole-rich cardamom oil has been shown to exhibit attenuation of oxidative stress, anti-inflammatory effects, acetylcholinesterase inhibition, BDNF upregulation, and reduction of amyloid pathology (Juergens, 2014; Kandikattu et al., 2017; Auti and Kulkarni, 2019, 2025). Supporting evidence were also available from *Elettaria cardamomum* studies indicating enhanced learning and memory, increased monoamine and glutathione levels, and reduced oxidative damage in experimental models (Abu-Taweel, 2018; Saini et al., 2021). Collectively, these mechanistic and preclinical findings provide a strong biological rationale for the observed cognitive effects of MA2-24.

However, this study was limited by its single-dose acute design, which restricts conclusions regarding the long-term efficacy, sustainability of cognitive benefits, and safety profile. Lack of biochemical marker data, relatively narrow population, and mixed cohort data are other limitations of the study, which warrant comprehensive future research.

## 5 Conclusion

A single dose of MA2-24, a full-spectrum aqueous extract of black cardamom fruits (*A. subulatum* Roxb.), demonstrated significant cognitive-enhancing effects across multiple domains, including attention, working memory, and processing speed, which helps in executive function. These effects were observed for 1- to 3-h post-dose and were further prolonged for 5 to 8 h when co-supplemented with caffeine, indicating a synergistic interaction. The results reflected significant treatment, time, and treatment-by-time effects, suggesting that MA2-24, alone or in combination with caffeine, can improve mental energy, alertness, and reduce cognitive fatigue. Thus, the present pilot study points to the potential of MA2-24 as a plant-based nootropic adjunct to enhance and sustain caffeine-mediated cognitive benefits and potentially mitigate post-caffeine performance decline. However, future research on the effect of long-term supplementation of MA2-24 and its underlying mechanisms, including effects on neurotransmission, neuroplasticity, and brain metabolism, is warranted to explore its potential for human supplementation.

## Data Availability

The original contributions presented in the study are included in the article/Supplementary material, further inquiries can be directed to the corresponding author.

## Footnotes

1
www.ncbi.nlm.nih.gov
