# Supplementary material for: A full-spectrum aqueous extract of black cardamom (Amomum subulatum) improves focus/alertness and executive function: a randomized, double-blinded, placebo- and active-controlled, comparative study in healthy working-class participants
Source: Front Neurosci. 2026 Apr 8;20:1786880. doi: 10.3389/fnins.2026.1786880 (PMC13100988; doi:10.3389/fnins.2026.1786880)
Supplement: Supplementary file 1 [file Table_1.docx]

**A full-spectrum aqueous extract of black cardamom (*Amomum subulatum*) improves focus/alertness and executive function:** **A randomized, double-blinded, placebo- and active-controlled, comparative study in healthy working-class participants**

Jestin V. Thomas^1^, Mohan M^2^, Syam S Das^3^, Nithyanandam Allimuthu^4^, Sheena Devasia^3^, Aneesa PA^5,7^, Krishnakumar Madhavamenon^3^, Baby Chakrapani PS*^5,^

**Supplementary data**

**Table S1.** Inclusion and exclusion criteria

| **Inclusion criteria**   1. Male and female working professionals aged between 35 and 65 years, inclusive. 2. Individuals with a BMI ranging from 18.5 kg/m² to 29.9 kg/m², inclusive. 3. Working professionals with a Fatigue Severity Scale (FSS) score greater than 4. 4. Those who are willing to maintain regular lifestyle, abstain from taking medications, alcohol, or caffeine as directed, and to comply with the study restrictions and requirements 5. Must refrain from vigorous physical activity for at least 12 hours before each study visit. 6. Female participants of reproductive age agree to use approved contraceptive methods and to have a negative urine pregnancy test at the screening visit 7. Participants must be versatile with the use of computers and should be willing to provide written informed consent |
| --- |
| **Exclusion criteria**   1. Individuals currently consuming any medications or substances that enhance cognition, energy, or mood, including alcohol, tobacco, or other central nervous system (CNS)-active drugs. 2. Individuals with known hypersensitivity or a history of allergic reactions to any component of the investigational products or herbals in general. 3. Individuals experiencing moderate to severe fatigue or diagnosed with chronic fatigue syndrome. 4. Patients with malignant diseases, end-stage organ conditions, or laboratory abnormalities deemed by the investigator to pose risk or interfere with study assessments. 5. Patients with uncontrolled metabolic conditions (e.g., diabetes, thyroid disorders), or severe chronic diseases or any condition that is not suitable as per investigator’s opinion 6. Individuals diagnosed with any psychiatric conditions other than mild stress, anxiety or depression, or with sleep disturbances, or those taking sleep aid medications. 7. Individuals with systolic blood pressure (SBP) >160 mmHg or diastolic blood pressure (DBP) >100 mmHg at the screening visit. 8. Those taking any medications such as anxiolytics, antidepressants, antipsychotics, anticonvulsants, antihypertensives, centrally acting corticosteroids, opioid pain relievers, hypnotics, or prescription sleep aids. Also excluded are individuals with a current or past history of drug and/or alcohol abuse. 9. Females who are pregnant, breastfeeding, planning pregnancy during the study period, or those with a positive urine pregnancy test at the Screening or Randomization visit. 10. Individuals who have received any investigational drug or used an investigational device within the 3 months prior to study enrollment. |

**Table S2:** Demographic and clinical characteristics of enrolled participants

| **Characteristics** | **Placebo** | **MA2-24** | **Caffeine** | **MA2-24+ Caffeine** |
| --- | --- | --- | --- | --- |
| Gender (Male/Female) | 17/7 | 16/8 | 12/12 | 14/10 |
| Age (Years) | 45.08 ± 6.3 | 45.29 ± 7.14 | 43.08 ± 6.4 | 42.79 ± 4.7 |
| Height (cm) | 166.71 ± 7.6 | 166.90 ± 7.3 | 165.06 ± 8.1 | 166.63 ± 8.7 |
| Weight (kg) | 68.65 ± 8.8 | 68.27 ± 9.9 | 68.16 ± 8.1 | 68.53 ± 12.2 |
| BMI (kg/m^2^) | 24.68 ± 2.6 | 24.44 ± 2.6 | 25.02 ± 2.4 | 24.51 ± 2.8 |
| Temp (°F) | 97.80 ± 0.5 | 97.90 ± 0.5 | 98.12 ± 0.4 | 98.0 ± 0.4 |
| Heart rate (bpm) | 81.46± 9.6 | 80.58 ± 10.0 | 80.96 ± 11.0 | 82.0 ± 9.2 |
| Systolic BP (mm/Hg) | 122.67 ± 9.5 | 123.58 ± 10.6 | 119.58 ± 7.8 | 118.38 ± 9.9 |
| Diastolic BP (mm/Hg) | 80.88 ± 8.0 | 82.33 ± 8.0 | 78.54 ± 8.6 | 79.42 ± 7.7 |

**Table S3:** Summary of participants’ vital signs recorded at baseline and at predefined time points

| **Characteristics** | **Temp (°F)** | **Heart rate (bpm)** | **Systolic BP (mm/Hg)** | **Diastolic BP (mm/Hg)** |
| --- | --- | --- | --- | --- |
| **Placebo** | | | | |
| Baseline | 97.84 ± 0.5 | 84.83 ± 9.2 | 120.83 ± 8.8 | 78.08 ± 8.5 |
| 1 h | 98.21 ± 0.3 | 82.46 ± 5.3 | 119.67 ± 8.0 | 78.0 ± 6.0 |
| 3 h | 98.23 ± 0.2 | 77.88 ± 5.4 | 120.50 ± 5.6 | 76.88 ± 6.2 |
| 5 h | 97.99 ± 0.3 | 79.88 ± 4.0 | 120.75 ± 5.7 | 78.42 ± 6.6 |
| 8 h | 98.15 ± 0.4 | 79.17 ± 4.4 | 118.88 ± 5.9 | 75.29 ± 4.5 |
| **MA2-24** | | | | |
| Baseline | 97.95 ± 0.4 | 83.25 ± 10.8 | 121.92 ± 8.1 | 79.25 ± 7.9 |
| 1 h | 98.29 ± 0.4 | 80.08 ± 6.3 | 120.42 ± 4.4 | 77.42 ± 6.0 |
| 3 h | 98.20 ± 0.4 | 77.58 ± 7.2 | 120.25 ± 6.3 | 75.75 ± 6.7 |
| 5 h | 98.05 ± 0.4 | 78.04 ± 5.8 | 120.13 ± 4.8 | 76.58 ± 5.6 |
| 8 h | 98.12 ± 0.3 | 77.79 ± 5.0 | 120.42 ± 5.4 | 77.38 ± 6.5 |
| **Caffeine** | | | | |
| Baseline | 97.83 ± 0.4 | 82.67 ± 11.5 | 122.75 ± 10.6 | 81.75 ± 6.8 |
| 1 h | 98.20 ± 0.4 | 78.63 ± 6.9 | 121.29 ± 7.1 | 80.21 ± 5.9 |
| 3 h | 98.20 ± 0.3 | 78.88 ± 6.4 | 120.83 ± 7.8 | 79.46 ± 6.4 |
| 5 h | 98.00 ± 0.3 | 79.25 ± 6.6 | 121.58 ± 7.0 | 77.83 ± 5.5 |
| 8 h | 98.15 ± 0.3 | 77.67 ± 5.4 | 120.75 ± 6.2 | 76.38 ± 6.8 |
| **MA2-24+Caffeine** | | | | |
| Baseline | 97.89 ± 0.6 | 84.42 ± 8.1 | 119.58 ± 9.2 | 78.75 ± 8.7 |
| 1 h | 98.02 ± 0.3 | 81.75 ± 5.6 | 119.58 ± 6.0 | 78.25 ± 5.8 |
| 3 h | 98.04 ± 0.3 | 76.71 ± 4.9 | 119.46 ± 5.6 | 77.88 ± 5.5 |
| 5 h | 97.98 ± 0.4 | 80.42 ± 5.9 | 119.63 ± 6.0 | 77.96 ± 4.4 |
| 8 h | 98.06 ± 0.3 | 78.25 ± 5.3 | 120.21 ± 5.3 | 77.50 ± 5.7 |
